# Evidence of a Strong Domestication Bottleneck in the Recently Cultivated New Zealand Endemic Root Crop, *Arthropodium cirratum* (Asparagaceae)

**DOI:** 10.1371/journal.pone.0152455

**Published:** 2016-03-24

**Authors:** Lara D. Shepherd, Peter J. de Lange, Simon Cox, Patricia A. McLenachan, Nick R. Roskruge, Peter J. Lockhart

**Affiliations:** 1 Museum of New Zealand Te Papa Tongarewa, Wellington, New Zealand; 2 School of Biological Sciences, Victoria University of Wellington, Wellington, New Zealand; 3 Institute of Fundamental Sciences, Massey University, Palmerston North, New Zealand; 4 Science and Capability Group, Terrestrial Ecosystems, Department of Conservation, Newton, Auckland, New Zealand; 5 Institute of Agriculture and Environment, Massey University, Palmerston North, New Zealand; Fordham University, UNITED STATES

## Abstract

We use chloroplast DNA sequencing to examine aspects of the pre-European Māori cultivation of an endemic New Zealand root crop, *Arthropodium cirratum* (rengarenga). Researching the early stages of domestication is not possible for the majority of crops, because their cultivation began many thousands of years ago and/or they have been substantially altered by modern breeding methods. We found high levels of genetic variation and structuring characterised the natural distribution of *A*. *cirratum*, while the translocated populations only retained low levels of this diversity, indicating a strong bottleneck even at the early stages of this species’ cultivation. The high structuring detected at four chloroplast loci within the natural *A*. *cirratum* range enabled the putative source(s) of the translocated populations to be identified as most likely located in the eastern Bay of Plenty/East Cape region. The high structuring within *A*. *cirratum* also has implications for the conservation of genetic diversity within this species, which has undergone recent declines in both its natural and translocated ranges.

## Introduction

The emergence of agriculture was one of the most important developments in human history and lead to significant cultural and environmental changes [1]. There are a diversity of definitions for domestication but here we follow Zeder’s definition [2] where it is considered a sustained mutualistic relationship in which one organism influences the reproduction and care of another organism in order to secure a more predictable supply of a resource of interest. Given the importance of domestication it is unsurprising that a considerable amount of research has investigated the processes involved in domestications. Of particular interest has been where, when and how many times domestication has taken place [3]. The majority of modern crops were first brought into cultivation many thousands of years ago, and this makes studying the early stages of their domestication challenging. Furthermore, much of our understanding of domestication comes from research on model crops in the grass family [4].

Most modern crops exhibit low genetic diversity compared to the wild relatives from which they were domesticated [1, 5–7]. However, the point in their domestication history at which this diversity was lost, and whether it occurred all at once or gradually over a long period of time is unclear [6, 8]. Although domestication is a continuum in the interdependency of people and their domesticates [2,6] there are two stages that have been identified in the domestication process when diversity can be lost [7]. Firstly, a domestication bottleneck occurs when a subset of the wild populations is brought into cultivation. Following this initial bottleneck, diversity can subsequently be lost thorough selective breeding for desirable traits during crop improvement (an improvement bottleneck). Diversity can also be gained following the initial domestication bottleneck, through gene flow from wild relatives [9].

Some studies have been able to examine the effect of the improvement bottleneck on genetic diversity (reviewed in [7]). However, examining the effect of domestication bottlenecks is more difficult for many species (1) because thousands of years may have elapsed since domestication was initiated, (2) because of introgression of genetic material through hybridization with wild relatives and (3) because of change in the distribution and extinction of natural populations, cultivation and interactions with wild relatives [10,11].

In this study we examine the extent of the domestication bottleneck in an endemic New Zealand plant species. New Zealand offers a unique opportunity to study recent domestication because it was the last substantial landmass to be colonised [12], about 700–800 years ago [13]. Pacific Islanders translocated and cultivated a large number of plant species around the Pacific region, with a focus on tree and root crops such as taro (*Colocasia esculenta*; Araceae), sweet potato or kumara (*Ipomoea batatas*; Convolvulaceae) and breadfruit (*Artocarpus altilis*; Moraceae) [14,15]. It is probable that Polynesian settlers introduced many of these tropical crops to New Zealand but that only a few survived owing to the cooler climate compared with the tropical Pacific Islands [15]. Those introduced crops that did survive failed to thrive except in the warmest regions. To compensate for the loss and variable yields of these introduced crops, Māori began to cultivate a number of plants they discovered in New Zealand for food, medicine and fibre [16]. These indigenous crops were a particularly important food source in southern areas of New Zealand where the cultivation of introduced sub-tropical plant food crops was marginal [15]. Because these species are endemic to New Zealand the onset of their cultivation must only date, at most, to 800 years ago when New Zealand was colonised [13].

One such plant is rengarenga (repihina-papa, maikaika, New Zealand rock lily; *Arthropodium cirratum* (G.Forst) R.Br.), a perennial evergreen lily-like herb in the Asparagaceae family. This species is endemic to New Zealand, where it grows primarily in coastal areas on rock outcrops, cliff edges and slips [17]. *Arthropodium cirratum* is insect pollinated but also uses delayed autonomous self-pollination, where selfing occurs once the chance to outcross has passed [18]. The seeds of *Arthropodium* have been suggested to disperse by gravity [19] or wind, with the funicles attached to seeds suggested to be a possible adaptation to wind dispersal [20].

**Fig 1:**
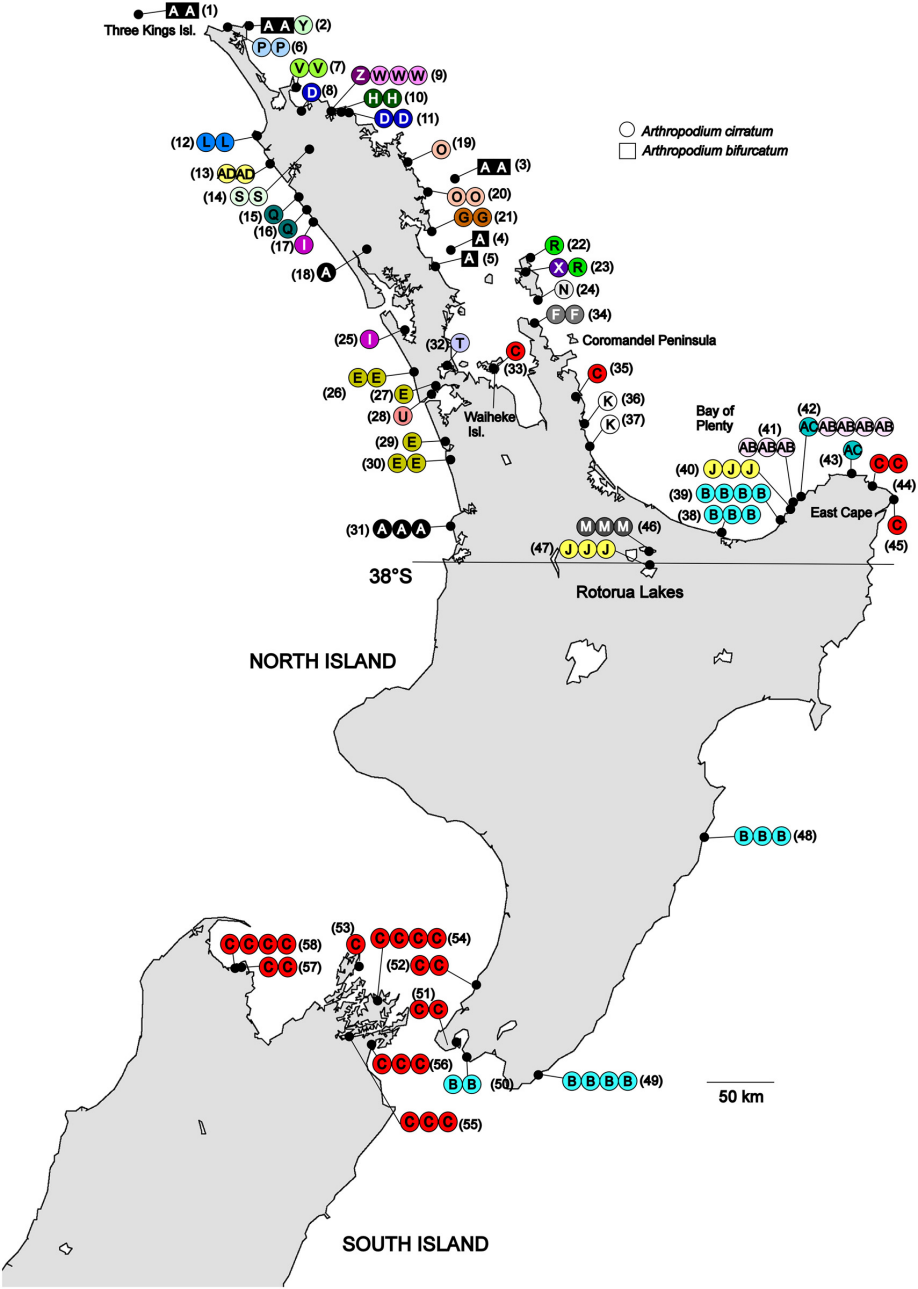
Geographic distribution of chloroplast haplotypes in *Arthropodium cirratum* and *A*. *bifurcatum*, based on alignment 1. Each individual sample is represented by a single coloured circle. The numbers in brackets are the sites’ identifying number; see S1 Table for more details. For *A*. *cirratum*, sites 1–47 are believed to be naturally occurring and sites 48–58 derive from translocations. The position of 38°S, an important biogeographic boundary in New Zealand, is indicated. Basemap supplied by Kahuroa.

The natural distribution of *A*. *cirratum* is thought to be north of around 38°S (Fig 1), which is the southern distributional limit for a number of plant species restricted to the northern North Island [17, 21, 22]. South of 38°S (Fig 1) it is usually associated with Māori archaeological sites, including gardens [17, 23]. Significantly it is often found growing with karaka (*Corynocarpus laevigatus*), an endemic tree known to have been cultivated by Māori [15, 24]. It has been speculated that these southern populations of *A*. *cirratum* are, or have established from, historical Māori cultivations [17, 23].

Colenso [25] recorded that the thick fleshy roots, which are up to 3 cm wide [26], were eaten. It was was considered a valuable food plant, despite the small yield, because it was hardy [27]. Harris and Te Whaiti [23] suggest *A*. *cirratum* may have been eaten in spring when fresh food was scarce and stored food supplies low. Its importance likely increased when the kumara crop yield was low. The roots were also used medicinally to treat boils and abscesses [28], and the plant had spiritual significance to Māori [23]. We do not know how this species was traditionally propagated by Māori, but it grows readily both from seed and through division [29].

Although no phenotypic changes between natural and translocated of *A*. *cirratum* populations are known, it is possible that characters that are not readily visible such as palatability and rhizome size were under selection. It has also been noted that the roots of plants propagated in cultivated soil grow much larger than those restricted to the rocky soils of cliffs [23].

The cultivation of *A*. *cirratum*, like that of many other pre-European crops grown by Māori, probably ceased once higher-yielding crops, such as potatoes and corn, were introduced by Europeans settlers in the late 18^th^ century [23]. *Arthropodium cirratum* is vulnerable to grazing by introduced mammalian herbivores [17, 23], and it has declined in some parts of its mainland range [17]. Within its translocated range, it is now commonly confined to cliffs that are inaccessible to browsing animals, particularly favouring those places also free from exotic slugs and snails.

Notwithstanding the diversity of definitions for domestication, we consider *A*. *cirratum* to be in the early stages of domestication following Zeder’s [2] definition (see above). Māori benefitted from translocating this species beyond its natural range by guaranteeing it as a readily-available food supply and *Arthropodium cirratum* has significantly increased its distribution.

In this study we examine the diversity at four chloroplast loci for *A*. *cirratum* in its natural and translocated range to address the following questions:

1. What is the chloroplast diversity and structuring in the natural populations of *A*. *cirratum*?
2. How many times was *A*. *cirratum* independently brought into cultivation and how much genetic diversity has been retained in the translocated sites?
3. Where are the locations of the initial source populations?
4. What are the relationships among translocated plants, and do these reflect Māori settlement routes and mobility?

## Materials and Methods

### Sample collection and DNA extraction

One hundred and nine *A*. *cirratum* specimens were collected from throughout its range (Fig 1). Eight samples of the closely-related *A*. *bifurcatum* Heenan, A.D.Mitch. et de Lange [17] and two samples of the third New Zealand species in the genus, *A*. *candidum* Raoul, were also included. For each specimen small pieces of leaf tissue were collected into silica-gel and a sample taken for a herbarium voucher (S1 Table). DNA was extracted from silica-gel dried leaf tissue using a modified CTAB extraction protocol (step 1, followed by steps 3–7 of Table 1 in [30]).

### PCR amplification and sequencing

For all specimens three DNA regions were initially PCR amplified: *rpl32–trnL*^*(UAG)*^, *3’ndhC-trnV*^*(UAC)*^, and *ndhH*-*rps15*. Primers for the first two regions were from [31]. Primers for the *ndhH*-*rps15* region (F- GGGTCCTGATAAACCCCAAT and R- AACGGCTGCTGGATTATTTG) were designed with Primer3 [32] from *A*. *bifurcatum* and *A*. *cirratum* chloroplast sequences generated by Illumina sequencing [33].

PCR amplification was performed in 10 μl reactions containing 1× PCR buffer (10 Mm Tris-HCl, 50 Mm KCl, pH 8.3; Roche Applied Science), 250 μmol dNTPs (Roche Applied Science), 1 M betaine, 10 ρmol of each primer and 1 U Taq DNA polymerase (Roche Applied Science). All loci were PCR amplified using the ‘slow and cold’ thermocycling protocol of [31]. PCR products were visualised by agarose gel electrophoresis. Amplification products were purified by digestion with 0.5 U shrimp alkaline phosphatase (SAP, USB Corp.) and 2.5 U exonuclease I (*Exo*I, USB Corp.) at 37°C for 30 minutes, followed by inactivation of the enzymes at 80°C for 15 minutes. Sequencing was performed with the ABI Prism Big Dye Terminator cycle sequencing kit version 3.1 on an ABI 3730 DNA sequencer (Massey University Genome Service, Palmerston North, New Zealand).

**Fig 2:**
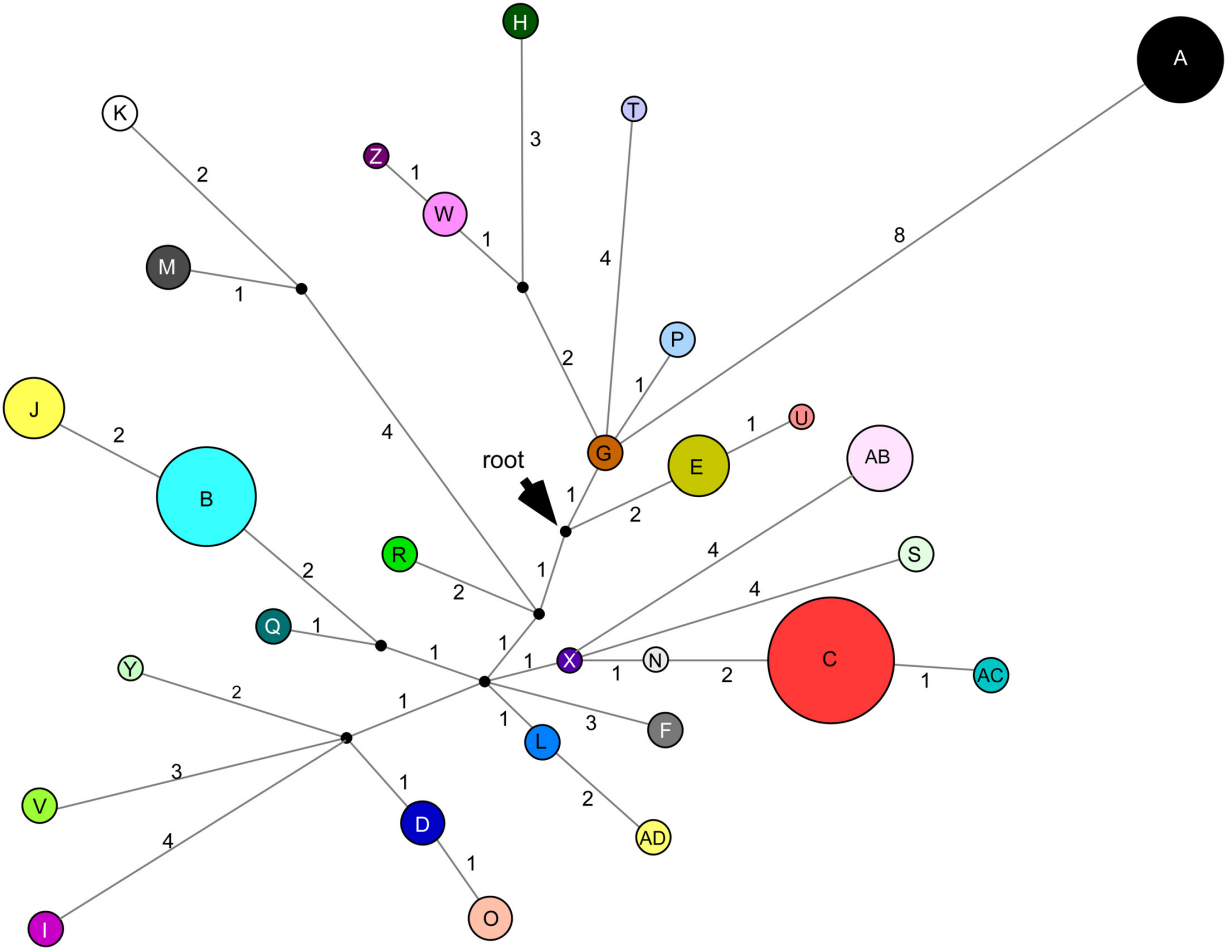
Median-joining haplotype network for the *Arthropodium cirratum* and *A*. *bifurcatum* chloroplast haplotypes. The size of each circle is proportional to haplotype frequency. Solid black circles correspond to missing intermediate haplotypes. The position of the root, where the outgroup *A*. *candidum* joins the network, is indicated. The number of mutational changes characterising each branch is shown.

Following the initial sequencing of these three loci, a comparatively common and reasonably widely distributed haplotype was determined (it included haplotypes C + AC + N, Fig 2). This haplotype was also detected in some of the translocated sites (sites 51–58). To further assess variation in plants bearing this haplotype, additional chloroplast sequences from a specimen from each of four sites (sites 24, 35, 42 and 56; Fig 1) were obtained using an Illumina MiSeq. The resulting sequences were mapped to the *A*. *bifurcatum* chloroplast genome from [33] in Geneious 8.04 (http://www.geneious.com [34]), and primer pairs were designed to three potentially variable target regions. These primer pairs were trialled on a subset of *A*. *cirratum* samples using the PCR conditions described above. The sequences generated with one of these primer pairs (F-GTTACAGAAGCGACCCCACA and R- GTGTGCGAGAACTTTGGCTC), which amplified a portion of the *psbA*-*matK* intergenic spacer, exhibited variation and sequenced cleanly so this locus was sequenced for the remaining samples.

Sequences were edited with Sequencer version 5.2.4 (Gene Codes Corp., Ann Arbor, MI, USA), and then aligned with Clustal X version 2.0 [35], with default parameters and manual alignment of large indels. Two alignments were produced: an alignment of the sequences from all four loci for *A*. *cirratum* and *A*. *bifurcatum* (alignment 1), and an alignment including sequences from the outgroup, *A*. *candidum*, in order to root the network (alignment 2). Sections of the *A*. *cirratum* and *A*. *bifurcatum rpl32–trnL*^(UAG)^ and *3’ndhC-trnV*^(UAC)^ regions were not able to be aligned to the outgroup sequences so only sequences of the *ndhH*-*rps15* region and *psbA*-*matK* intergenic spacer were used for alignment 2. For all analyses, indels were recoded as single events.

### Genetic diversity and structuring

Median-joining networks [36] were produced for both alignments using Network v4.613 (www.fluxus-engineering.com). Transitions and transversions were equally weighted.

The remaining analyses were calculated for *A*. *cirratum* using alignment 1. Nucleotide (π) and haplotype (*h*) diversity were calculated for *A*. *cirratum* (all samples) using Arlequin 3.5.1.2 [37]. The measures of genetic differentiation *G*_ST_ and *N*_ST_ were calculated both for all *A*. *cirratum* samples and for only the natural populations (sites 5 to 47) with the program SPADS 1.0 [38]. *G*_ST_ is calculated using only haplotype frequency data, whereas *N*_ST_ takes the relationships between haplotypes into account. *G*_ST_ and *N*_ST_ were compared using a permutation test with 1000 permutations. If *N*_ST_ is significantly greater than *G*_ST_, it suggests the presence of phylogeographic structuring with closely related haplotypes more likely to occur together in the same site [39].

The geographic structure of the genetic variation in the natural *A*. *cirratum* (sites 5 to 47) was examined by spatial analysis of molecular variance (SAMOVA [40]), implemented in SPADS 1.0 [38]. SAMOVA defines groups of populations by maximizing the proportion of the total genetic variance due to differences between groups of populations (F_CT_). The number of groups (*K*) was set to vary between 2 and 15, with 10 000 iterations and 10 repetitions. The optimal *K* was selected by choosing the highest F_CT_ where no groups comprised of samples from a single site.

We tested for isolation by distance by performing Mantel tests between *F*_ST_ and geographic distances in Arlequin 3.5.1.2 [37]. Two geographic distance matrices were tested: straight line geographic distances, calculated using the Geographic Distance Matrix Generator [41], and minimum coastline distance, measured from a map. Significance was tested with 1000 permutations.

### Demographic history of natural *Arthropodium cirratum*

The demographic history of *Arthropodium cirratum* within its natural distribution was investigated using several methods. In order to detect departure from neutral expectations, the neutrality tests Fu’s F_S_ [42] and Ramos-Onsins and Rozas’ R_2_ statistic [43] were calculated with DnaSP 5.10 [44]. These two tests have been found to be among the most powerful for detecting population growth [44]. Fu’s F_S_ is based on the probability of recovering a number of haplotypes greater or equal to the observed number of samples drawn from a constant-sized population [42]. Ramos-Onsins and Rozas’ R_2_ statistic is based on the difference between the number of singleton mutations and the average number of nucleotide differences [43]. The significance of Fu’s F_S_ and Ramos-Onsins and Rozas’ R_2_ were tested with 1000 coalescent simulations using DnaSP. A mismatch distribution of the pairwise genetic differences between haplotypes [45] was conducted using Arlequin 3.5.1.2 to test for the non-random distribution of coalescent events. Pairwise differences typically form two main patterns: (1) multimodal distributions are consistent with demographic stability, (2) unimodal distributions indicate a recent population expansion or population bottleneck [46]. An expected distribution under a model of sudden demographic expansion was generated with 1000 parametric bootstrap replicates [47]. The sum of squared deviations (*SSD*) [37] between the observed and expected mismatch distributions and Harpending’s raggedness index (*Hri*) [48], which quantifies the smoothness of the mismatch distribution were calculated to test the fit of the sudden expansion model. Small *Hri* values are typical of an expanding population whereas higher values are observed among stationary or bottlenecked populations [48].

## Results

The aligned length across all four loci for the *Arthropodium cirratum* and *A*. *bifurcatum* sequences (= alignment 1) was 3341 bp (*rpl32–trnL*^(UAG)^—902 bp; *3’ndhC-trnV*^(UAC)^—1826 bp; *ndhH*-*rps15*–773 bp; *psbA*-*matK—*740 bp). The sequence alignment from these two species contained 51 nucleotide substitutions and 13 indel events, ranging from 1 bp to 20 bp, defining 29 haplotypes (haplotypes A to Z and AB to AD, S1 Fig; GenBank Accession numbers in S1 Table; sequence alignments in S4, S5, S6 and S7 Figs).

### Genetic diversity and structuring

Only a single haplotype, A, was detected from *A*. *bifurcatum* (Fig 1). *Arthropodium cirratum* showed much greater diversity with 29 haplotypes recorded across the four loci, including haplotype A. The haplotype and nucleotide diversities of *A*. *cirratum* were 0.9105 ± 0.0175 and 0.0023 ± 0.00119, respectively.

The relationships between the 29 haplotypes are shown in the median-joining network in Fig 2. The maximum number of mutational steps between haplotypes in the network was 16. The network was rooted with alignment 2, which contained the outgroup *A*. *candidum* sequences and was 3160 bp in length. The two *A*. *candidum* sequences were identical to each other and joined the *A*. *cirratum*/*A*. *bifurcatum* network at an undetected intermediate haplotype most closely related to haplotypes E and G.

The distributions of the haplotypes are shown in Fig 1. Six haplotypes (N, T, U, X, Y, and Z) were each only detected from a single individual. Most of the remaining haplotypes had very restricted distributions and were only found in one or several geographically-close sites. Excluding the putative translocated plants, the largest geographic distance between sites with the same haplotype was ~500 km for haplotype A (site 1—*A*. *bifurcatum* to site 31—*A*. *cirratum*). For haplotypes only found within *A*. *cirratum*, haplotype C had the widest distribution (site 33 to site 45), a distance of ~300 km.

Population differentiation was very high for both the analyses with all the *A*. *cirratum* samples and for the natural sites only (all *A*. *cirratum*: *G*_ST_ = 0.936, *N*_ST_ = 0.997; natural *A*. *cirratum* only: *G*_ST_ = 0.912, *N*_ST_ = 0.961). For both datasets, *G*_ST_ was significantly higher than *N*_ST_ (all samples P < 0.006; natural samples P < 0.004), suggesting there was some phylogeographic component to the structuring.

**Fig 3:**
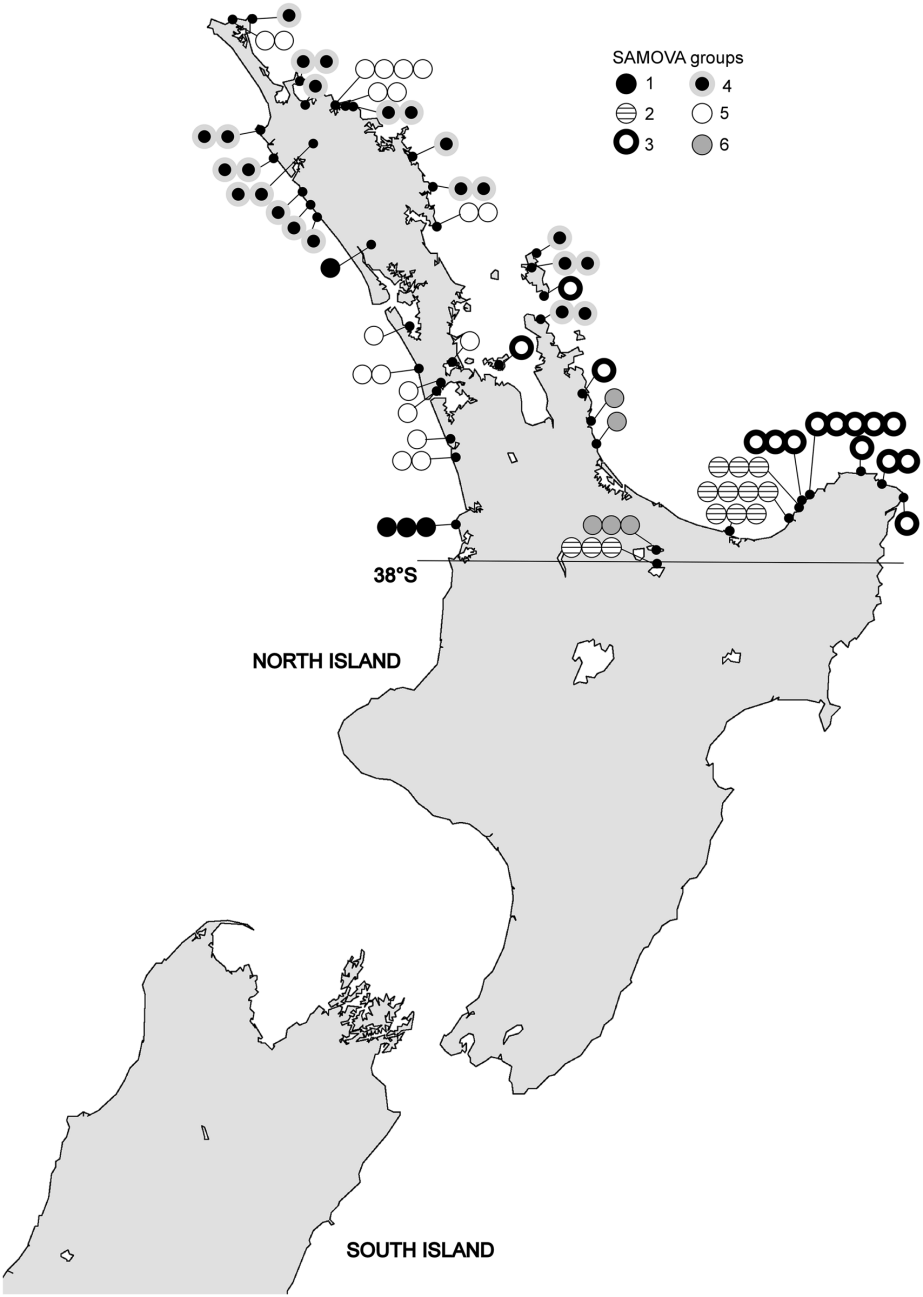
The distribution of the *Arthropodium cirratum* SAMOVA clusters for K = 6. Basemap supplied by Kahuroa.

For the SAMOVA analysis of the natural *A*. *cirratum* sites the gradual increase in F_CT_ values (S2 Fig) made it difficult to unambiguously identify the optimal *K*. However, there was a local maxima at *K* = 6 (F_CT_ = 0.637) and this was the highest F_CT_ when no groupings contained single sites. The distributions of the six groupings are shown in Fig 3. Only one of these groups contained only a single haplotype (Group 1 –haplotype A). The geographic ranges of all of the groups differed but most overlapped with the distribution of at least one other group.

For the natural *A*. *cirratum* sites, Fu’s F_S_ was negative and significant (F_S_ = -7.311, p = 0.021), indicating an excess of low frequency haplotypes suggesting population expansion In contrast, Ramos-Onsins and Rozas’ R_2_ was not significant (R_2_ = 0.0551, p = 0.062).

The mismatch distribution was unimodal (S3 Fig). The *SSD* and *Hri* were not significant (*SSD* = 0.002, p > 0.3; *Hri* = 0.009, p > 0.3); therefore, the sudden population expansion model could not be rejected. The small *Hri* value provided further support for a demographic expansion.

The Mantel test revealed weak but significant positive relationships between straight line geographic and genetic distance (r = 0.151; P<0.01) indicating isolation-by-distance. This pattern strengthened when coastline distance was used (r = 0.181; P<0.01).

### Translocation history of *Arthropodium cirratum*

Of the 29 haplotypes detected in *A*. *cirratum* only two (B and C) were found in the sites that are considered derived from Māori cultivation (sites 46–58). In the median-joining network these two haplotypes are distantly related and differ by seven mutational changes. Within the translocated populations haplotype B was detected from the east coast of the North Island (sites 48 and 49) and the south-west of the North Island (site 50). Within the natural range this haplotype was detected only in two adjacent sites in the eastern Bay of Plenty (sites 38 and 39), indicating this region may be the source for these translocated populations. Haplotype C was detected in the south-western North Island (sites 51 and 52) and was the only haplotype detected in the South Island (sites 53 to 58). The comparatively wide range of haplotype C in the natural distribution of *A*. *cirratum* (sites 33, 35, 44 and 45) prevented the identification of the precise source for the translocated populations containing haplotype C.

## Discussion

### Phylogeography of *Arthropodium cirratum* within its natural range

The most divergent haplotype detected within *Arthropodium cirratum*, haplotype A, differed from the remaining haplotypes by at least eight mutational changes and was also shared with *A*. *bifurcatum*. Shared haplotypes among species can be attributed to gene flow through hybridization or incomplete lineage sorting [49]. Neither of the two *A*. *cirratum* sites in which haplotype A occurred are in close proximity to any *A*. *bifurcatum*. Where the two species are sympatric there is no evidence of hybrids or clines of variation [17]. Therefore, the sharing of haplotype A between these two species is likely to be a consequence of a more ancient hybridization event or lineage sorting. Further study with nuclear markers is required to distinguish between these hypotheses.

Within its putative natural range *A*. *cirratum* demonstrated a very high level of chloroplast structuring (cf. [50]). The genetic differentiation between adjacent sites likely results from restricted seed dispersal. The lack of chloroplast haplotype sharing, the non-star-like network and significant isolation by distance indicates prolonged historical isolation of populations. However, the significant negative Fu’s F_S_ and the mismatch distribution indicate that *A*. *cirratum* has undergone past population expansion within its natural range. It should be noted that Fu’s F_s_ calculation assume that sequences are drawn from a panmictic (non-structured) population. Our samples clearly derive from structured populations but simulations have shown that population structuring results in more positive values of these statistics [51], thus decreasing the signal of expansion. We suggest that the observed phylogeographic pattern for *A*. *cirratum* might be the consequence of an initial population expansion, followed by population bottlenecks and isolation leading to genetically distinct populations.

The structuring observed in *A*. *cirratum* has implications for conservation, with recent declines reported for a number of mainland populations and conservation management of representative populations recommended [17]. Our results suggest a large number of populations would need to be conserved in order to encompass the detected chloroplast diversity.

### Loss of diversity of cultivated *Arthropodium cirratum*

Only 2 of the 29 haplotypes detected in *A*. *cirratum* were found in the southern populations, indicating that, even at this early stage of domestication, considerable chloroplast diversity has been lost. Studies of both perennial and annual fruit crops showed high levels of diversity (91.4% and 74.1%, respectively), albeit calculated with nuclear markers, were maintained through domestication bottlenecks [7]. Chloroplast DNA has a smaller effective population size than nuclear DNA because it is haploid and uniparentally inherited. Therefore, a greater loss of chloroplast diversity than nuclear diversity is expected during a bottleneck [52]. Analysis of *A*. *cirratum* with nuclear DNA markers would permit a more direct comparision with these studies [7].

In addition to the type of genetic marker examined, the extent of the genetic diversity lost through a domestication bottleneck depends on many factors, including the level of genetic structuring in the wild populations, the number of times crops were independently domesticated (single versus multiple events), the size of the area over which domestication occurred, the extent of introgression from wild relatives post-domestication [53] and the mating system and mode of reproduction, e.g. by seed or vegetative means [54].

The low chloroplast diversity retained in the translocated populations of *A*. *cirratum* likely results from a combination of factors including (1) the very high chloroplast structuring within the natural distribution of *A*. *cirratum*, which likely results from its low seed dispersal abilities, (2) the narrow area from which plants were sourced for cultivation (see Sources of cultivated *Arthropodium cirratum* below) and (3) the physical isolation of the translocated populations from the natural populations, which has prevented gene flow from introducing additional wild diversity into the cultivated populations.

Analysis of *A*. *cirratum* with nuclear DNA markers may be able to determine whether this species was propagated by seed or vegetatively, with vegetative reproduction likely to lead to lower variation in the cultivated populations. However, the propensity for selfing in this species may also lead to low diversity. Nuclear data may also be able to distinguish whether the translocated populations were moved south in a stepping stone colonisation pattern e.g., was the population at Tora (site 49) sourced from Kairakau (site 48) or was it an independent translocation from the natural range?

### Sources of cultivated *Arthropodium cirratum*

It is possible that the two translocated haplotypes were introduced from a single source population (= a single domestication event). However, this seems unlikely because this source population would have had to have been more diverse than any extant population we sampled. Alternatively, the two haplotypes derive from multiple, genetically-distinct, source populations. Regardless of whether the cultivated plants derive from single or multiple sources populations it is likely the source(s) were located in the eastern Bay of Plenty/East Cape region, where haplotypes B and C both occur. However, sites 33 and 35, from outside this region, cannot be excluded as possible sources of haplotype C with our data, although it is also possible that they too derive from translocation from the East Cape region. Future analyses with nuclear markers may provide a clearer picture of the number of times *A*. *cirratum* has been brought into cultivation and the initial source locations.

The coastal terraces of the eastern Bay of Plenty and northern East Cape have been recognised as an area used extensively by Māori for gardening [55]. Why this region might have been the source of cultivated populations translocated further south in unclear. *Arthropodium cirratum* may have first been recognised as a food source by iwi (Māori tribes) in this region or plants from this location had some desirable characteristics, such as palatability, starch content or root size. A comprehensive study of the root morphologies of natural and cultivated *A*. *cirratum* plants, which has not been done, may indicate whether any morphological characters were under selection, as well as provide additional evidence for the source of the cultivated populations.

A previous study of an endemic New Zealand plant species, *Hebe speciosa*, by Armstrong & de Lange [56] examined the origin of populations of this species in the northern South Island using AFLP analysis. This species is largely coastal and occurs naturally along the north-western coastline of the North Island. Armstrong & de Lange [54] concluded from their analyses that the South Island population of *H*. *speciosa* at Titrangi Bay in the Marlborough Sounds (site 54, Fig 1) was translocated by Māori from the Hokianga Harbour in the northern North Island, probably for ornamental purposes. Both sites were important regional trade locations. Our results for *A*. *cirratum* do not show links between these two regions. The two haplotypes we detected in the Hokianga region (haplotypes Q and S) were not found in Titirangi Bay, or any other translocated population. Further molecular studies of other species translocated by Māori such as karaka (*Corynocarpus laevigatus* [15, 24]) and New Zealand flax (*Phormium tenax* [57]) may provide a more detailed picture of the multiple trade routes that were used by Māori. Comparisons should also be made to known trade routes within the extensive Māori trade system for obsidian and greenstone. These valuable materials, used for making tools, were translocated throughout New Zealand. Obsidian from Mayor Island in the Bay of Plenty, was moved as far afield as the Kermadec Islands, Norfolk Island, Auckland Islands and the Chatham Islands [58, 59, 60, 61, 62, 63, 64, 65, 66].

For the *A*. *cirratum* translocated populations, the lack of overlap in the distributions of haplotypes B and C may suggest that once plants were established there was limited movement of plants between gardens, otherwise sites with both haplotype B and C might be expected. Nuclear markers may be able to examine this hypothesis further.

It is possible that *A*. *cirratum* at sites 46 and 47 from the Rotorua Lakes also derive from translocation. Some plant species in this region, including *A*. *cirratum*, have been suggested to have been planted by Māori [67]. However, there are also many typically coastal species occuring naturally inland here. If the *A*. *cirratum* at the Rotorua Lakes did derive from translocation then the number of translocated haplotypes would increase to four and these two additional haplotypes would also be consistent with a Bay of Plenty/East Cape origin. One of the haplotypes, Haplotype M, was not detected elsewhere but is most closely related to haplotype K (sites 36 and 37; Fig 3), found nearby in the western Bay of Plenty. The other haplotype from the Rotorua Lakes, Haplotype J, was also detected at Haparapara River (site 40), eastern Bay of Plenty.

### Phylogeography of *Arthropodium bifurcatum*

*Arthropodium bifurcatum* was only recently recognized as a species distinct from *A*. *cirratum* [17]. *Arthropodium bifurcatum* mainly occurs on the coast of offshore islands but is sympatric on the mainland coast with *A*. *cirratum* in a few localities [17]. No diversity was detected in *A*. *bifurcatum* at the four chloroplast loci sequenced. It is perhaps surprising that *A*. *bifurcatum* from the Three Kings Islands was not distinct. This island group is home to a number of endemic species. Among species shared with the mainland a number of plants and insect populations on the Three Kings Island have been found to be genetically distinct from their mainland counterparts [24, 68]. Buckley & Leschen [68] used molecular dating to estimate divergence times between insect lineages on the Three Kings Islands and their sister groups from the rest of New Zealand. From their results they suggested that there was no land connection between the Three Kings Islands and mainland New Zealand during the Pleistocene. At present there is no reliable method by which to date the divergences within *Arthropodium* (no substitution rates have been calculated in the Asparagaceae for any of the four chloroplast loci we sequenced and the relationship of a tentative mid-Miocene *Arthropodium* fossil to contemporary species is unknown [69]). However, the lack of diversity in the sequences we obtained from *A*. *bifurcatum* suggests it is unlikely that the Three Kings Islands population of *A*. *bifurcatum* diverged from the other populations of this species prior to the Pleistocene.

## Supporting Information

S1 Fig Variable nucleotide positions at four chloroplast loci in *Arthropodium cirratum* based on alignment 1. Sequences are compared to the sequence in *A*. *bifurcatum* (haplotype A). The nucleotide position in the alignment of *A*. *cirratum* and *A*. *bifurcatum* sequences is shown at the top, and deletions are indicated by:.
(PDF)

S2 Fig Plot of the fixation indices (F_CT_) obtained from SAMOVA. Analysis was performed only on the natural populations of *Arthropodium cirratum*.
(TIF)

S3 Fig Mismatch distribution of *Arthropodium cirratum* haplotypes. The plot shows the frequencies of all pairwise differences for the *Arthropodium cirratum* haplotypes from the natural distribution (bars). Frequencies simulated under a sudden expansion model are also shown (line).
(TIF)

S4 Fig Fasta formatted sequence alignment of the *3’ndhC-trnV*^*(UAC)*^ region. (TXT)

S5 Fig Fasta formatted sequence alignment of the *ndhH*-*rps15* region. (TXT)

S6 Fig Fasta formatted sequence alignment of the *rpl32–trnL*^*(UAG)*^ region. (TXT)

S7 Fig Fasta formatted sequence alignment of the *psbA*-*matK* intergenic spacer. (TXT)

S1 Table Details of the *Arthropodium* samples. Herbarium voucher number, source population details, chloroplast haplotype and GenBank numbers. Sample localities in bold for *A*. *cirratum* are within the putative natural distribution of this species.
(DOCX)
