# Supplementary material for: Evidence of a Strong Domestication Bottleneck in the Recently Cultivated New Zealand Endemic Root Crop, Arthropodium cirratum (Asparagaceae)
Source: PLoS One. 2016 Mar 24;11(3):e0152455. doi: 10.1371/journal.pone.0152455 (PMC4806853; doi:10.1371/journal.pone.0152455)
Supplement: S1 Fig — Sequences are compared to the sequence in A. bifurcatum (haplotype A). The nucleotide position in the alignment of A. cirratum and A. bifurcatum sequences is shown at the top, and deletions are indicated by:. (PDF) [file pone.0152455.s001.pdf]

```
   ndhH–rps15                                 rpl32–trnL(UAG)                                  3’ndhC–trnV(UAC)    psbA–matK
    123456777     111112223333333333333333333333333333344444444444444444444444455566667777768   11234555555577789  1223445667
   4513285056 777744788229000000000011111111112222222671777777777788888888889911103660000080 6809266111226612982 59554998150
   1992312266 012329979349012345678901234567890123456624012345678901234567892312438890123483 7877366789013444401 72567459097
A  TGAGATTACC :::CAGGTGCATCAATATTAGTTAGTCAATATTAGTTAGCGA::::::::::::::::::::AAAAAACGTAATGACA AGACCAC:::::CTTATTG CGT:CGCGGAC
B  C...GCG... TT.:.....................::::::::::::::..........................TTT.......... ..C...........G...T A..........
C  C....CG... .........................::::::::::::::..........................TTT.......... ..C.A.A.......G...T A.:..A.....
D  C....CG... ...........::::::::::::::::::::::::::::..........................TTT.........T ..C...........G...T A..........
E  C....CG... .........................::::::::::::::.......................:..TTT.......... ..C.........A.G.... ...........
F  C....CG T.. ........................::::::::::::::..........................TTT.......... ..C...........G...T A.........T
G  C....TG... .........................::::::::::::::..........................TTT.......... ..C...........G.... ...........
H  C.GC.TG... ......T..................::::::::::::::..C.......................TTT.......... ..C...........G.... .........C.
I  C....CG.T. .......A...::::::::::::::::::::::::::::.A........................TTT.......... G.C...........G...T A..........
J  C...GCG... TTT:.....................::::::::::::::..........................TTT.......... ..C...........G...T A.......A..
K  C....CG... ........A..............................T.........................TTTG......... ..C...........G...T ..:....A...
L  C....CG... .........A...............::::::::::::::..........................TTT.......... ..C...........G...T A..T.......
M  C....CG... ........A................::::::::::::::T.........................TTT.......... .TC...........G...T ..:....A...
N  C....CG... .........................::::::::::::::..........................TTT.......... ..C.A.A.......G...T A..........
O  C....CG... ...........::::::::::::::::::::::::::::...TTAATTTTCTTTTCACAATA...TTT.........T ..C...........G...T A..........
P  C....TG... .........................::::::::::::::........................G.TTT.......... ..C...........G.... ...........
Q  C...GCG... .........................::::::::::::::..........................TTT.......... ..C...........G...T A..........
R  C....CG... ....CT...................::::::::::::::..........................TTT.......... ..C...........G...T ...........
S  C....CG... .........................::::::::::::::.......................:.:TTT........A. ..CA..........G...T A..........
T  C....CG... ..........C..............::::::::::::::..........................TTT.......... ..C..T.......GG.... ....A......
U  C....CG... .........................::::::::::::::.......................:..TTT.......... ..C.........A.G.G.. ...........
V  C....CG... .........................::::::::::::::..........................TTT.......... .T............GG..T A..........
W  C....TG... .........................::::::::::::::..C.......................TTT.......... ..C...........G.C.. .........C.
X  C....CG.. ..........................::::::::::::::..........................TTT.......... ..C...A.......G...T A..........
Y  C....CG..A ...........::::::::::::::::::::::::::::..........................TTT.......... ..C...........G...T AC.........
Z  C....TG... .........................::::::::::::::..C.......................TTT.......... ..C...........G.CC. .........C.
AB CA...CG... .........................::::::::::::::..........................TTT.:::::::.. ..C...AGAAAT..G...T A.:........
AC C....CG... .........................::::::::::::::..........................TTT.......... ..C.A.A.......G...T ..:..A.....
AD C....CG... .........A...............::::::::::::::..........................TTT.......... ..C...........G...T A..T..A....
```
