# Supplementary figures and images for: Evidence of a Strong Domestication Bottleneck in the Recently Cultivated New Zealand Endemic Root Crop, Arthropodium cirratum (Asparagaceae)
Source: PLoS One. 2016 Mar 24;11(3):e0152455. doi: 10.1371/journal.pone.0152455 (PMC4806853; doi:10.1371/journal.pone.0152455)

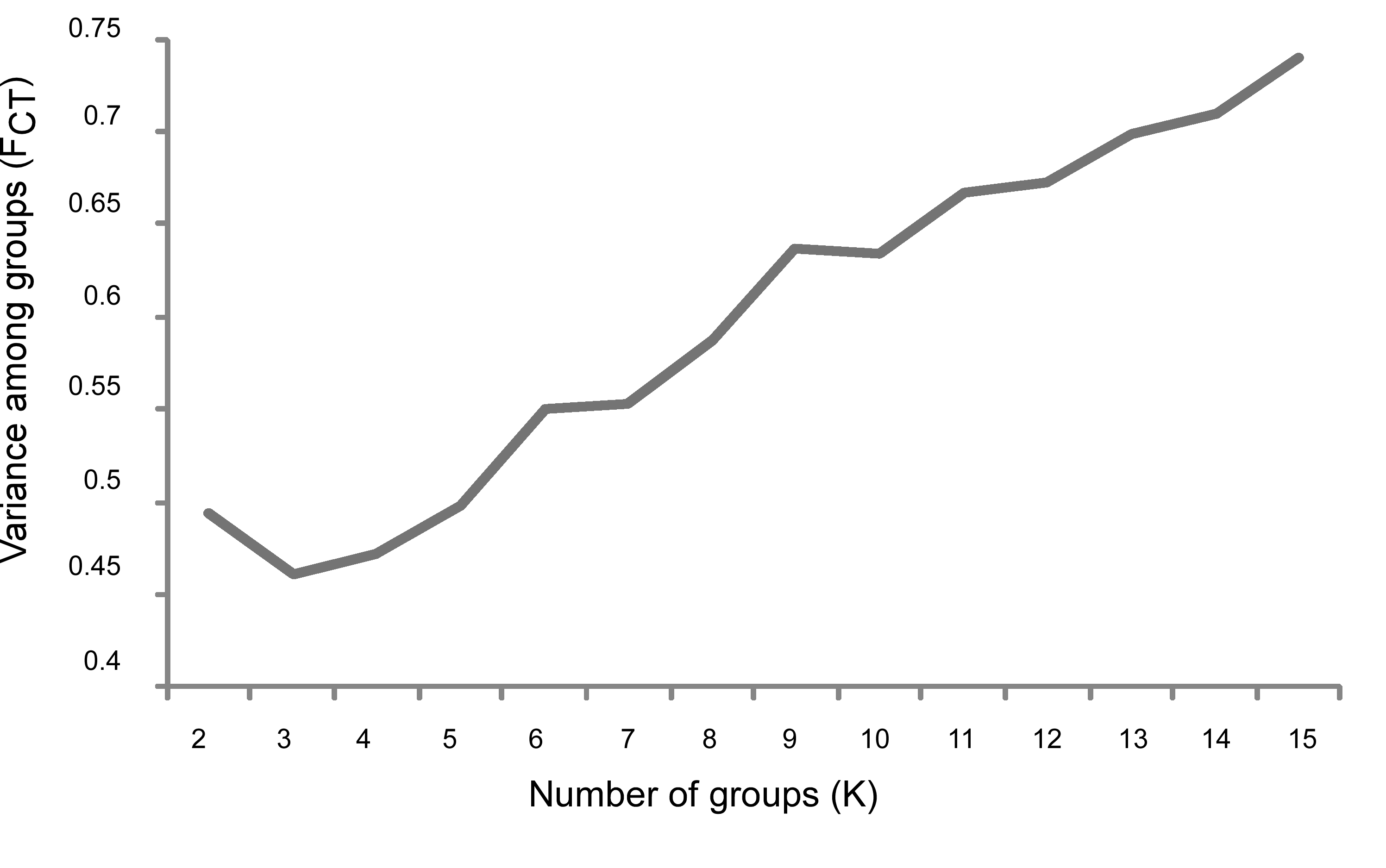

Supplement: S2 Fig — Analysis was performed only on the natural populations of Arthropodium cirratum. (TIF) [file pone.0152455.s002.tif]
