# Supplementary material for: Evidence of a Strong Domestication Bottleneck in the Recently Cultivated New Zealand Endemic Root Crop, Arthropodium cirratum (Asparagaceae)
Source: PLoS One. 2016 Mar 24;11(3):e0152455. doi: 10.1371/journal.pone.0152455 (PMC4806853; doi:10.1371/journal.pone.0152455)
Supplement: S1 Table — Herbarium voucher number, source population details, chloroplast haplotype and GenBank numbers. Sample localities in bold for A. cirratum are within the putative natural distribution of this species. (DOCX) [file pone.0152455.s008.docx]

**Table S1. Details of the *Arthropodium* samples, with representative herbarium voucher numbers, source population details, chloroplast haplotype and GenBank numbers.** Sample localities in bold for *A. cirratum* are within the putative natural distribution of this species.

| Species | Site identifier | Location | Latitude, Longitude | Voucher | Haplotype | GenBank Accession Numbers | | | |
| --- | --- | --- | --- | --- | --- | --- | --- | --- | --- |
|  |  |  |  |  |  | *ndhH*-*rps15* region | *rpl32–trnL^(UAG)^* region | *3’ndhC-trnV^(UAC )^* region | *psbA*-*matK* region |
| *A. bifurcatum* | 1 | Great Island, Three Kings | -34.1575, 172.1387 | WELT SP103534 | A | KT949661 | KT949425 | KT949780 | KT949542 |
| *A. bifurcatum* | 1 | In cultivation, ex. Three Kings Islands | -34.1693, 172.1045 | WELT SP103441 | A | KT949656 | KT949420 | KT949775 | KT949537 |
| *A. bifurcatum* | 2 | Surville Cliffs | -34.3920, 173.0188 | AK 309832 | A | KT949663 | KT949427 | KT949782 | KT949544 |
| *A. bifurcatum* | 2 | In cultivation, ex. Surville Cliffs | -34.3956, 173.0124 | WELT SP103511 | A | KT949658 | KT949422 | KT949777 | KT949539 |
| *A. bifurcatum* | 3 | In cultivation, ex. Poor Knights Islands | -35.4688, 174.7365 | WELT SP103513 | A | KT949659 | KT949423 | KT949778 | KT949540 |
| *A. bifurcatum* | 3 | In cultivation, ex. Poor Knights Islands | -35.4688, 174.7365 | WELT SP103512 | A | KT949657 | KT949421 | KT949776 | KT949538 |
| *A. bifurcatum* | 4 | In cultivation, ex. Hen Island | -35.8917, 174.7274 | WELT SP103440 | A | KT949655 | KT949419 | KT949774 | KT949536 |
| *A. bifurcatum* | 5 | Bream Tail,  Northland | -36.0604, 174.5899 | WELT SP103516 | A | KT949660 | KT949424 | KT949779 | KT949541 |
| *A. cirratum* | 2 | **Surville Cliffs, Northland** | -34.3956, 173.0124 | WELT SP103437 | Y | KT949759 | KT949523 | KT949878 | KT949640 |
| *A. cirratum* | 6 | **Te Paki, Northland** | -34.3956, 173.0124 | WELT SP103510 | P | KT949743 | KT949624 | KT949862 | KT949624 |
| *A. cirratum* | 6 | **Te Paki, Northland** | -34.3956, 173.0124 | WELT SP103510 | P | KT949744 | KT949508 | KT949863 | KT949625 |
| *A. cirratum* | 7 | **Whatuwhiwhi, Northland** | -34.8827, 173.3992 | WELT SP103529 | V | KT949754 | KT949518 | KT949873 | KT949635 |
| *A. cirratum* | 7 | **Whatuwhiwhi, Northland** | -34.8827, 173.3992 | WELT SP103529 | V | KT949753 | KT949517 | KT949872 | KT949634 |
| *A. cirratum* | 8 | **Taipa, Northland** | -34.9895, 173.4727 | WELT SP103433 | D | KT949711 | KT949475 | KT949830 | KT949592 |
| *A. cirratum* | 9 | **Ranfurly Bay Scenic Reserve, Northland** | -35.0169,  173.7238 | WELT SP103383 | W | KT949755 | KT949519 | KT949874 | KT949636 |
| *A. cirratum* | 9 | **Ranfurly Bay Scenic Reserve, Northland** | -35.0169,  173.7238 | WELT SP103370 | W | KT949756 | KT949520 | KT949875 | KT949637 |
| *A. cirratum* | 9 | **Ranfurly Bay Scenic Reserve, Northland** | -35.0169,  173.7238 | WELT SP103383 | W | KT949757 | KT949521 | KT949876 | KT949638 |
| *A. cirratum* | 9 | **Ranfurly Bay Scenic Reserve, Northland** | -35.0169,  173.7238 | WELT SP103383 | Z | KT949763 | KT949524 | KT949879 | KT949641 |
| *A. cirratum* | 10 | **Tauranga Bay, Northland** | -35.0095, 173.7801 | WELT SP103525 | H | KT949722 | KT949486 | KT949841 | KT949603 |
| *A. cirratum* | 10 | **Tauranga Bay, Northland** | -35.0095, 173.7801 | WELT SP103525 | H | KT949723 | KT949487 | KT949842 | KT949604 |
| *A. cirratum* | 11 | **Mahinepua Peninsula Scenic Reserve, Northland** | -34.9974, 173.8491 | WELT SP103533 | D | KT949709 | KT949473 | KT949828 | KT949590 |
| *A. cirratum* | 11 | **Mahinepua Peninsula Scenic Reserve, Northland** | -34.9974, 173.8491 | WELT SP103533 | D | KT949710 | KT949474 | KT949829 | KT949591 |
| *A. cirratum* | 12 | **Ahipara, Northland** | -35.1866, 173.1224 | WELT SP103538 | L | KT949734 | KT949498 | KT949853 | KT949615 |
| *A. cirratum* | 12 | **Ahipara, Northland** | -35.1866, 173.1224 | WELT SP103538 | L | KT949735 | KT949499 | KT949854 | KT949616 |
| *A. cirratum* | 13 | **Whangape Ridge, Northland** | -35.3651, 173.2187 | WELT SP103517 | AD | KT949770 | KT949534 | KT949889 | KT949651 |
| *A. cirratum* | 13 | **Whangape Ridge, Northland** | -35.3651, 173.2187 | WELT SP103517 | AD | KT949771 | KT949535 | KT949890 | KT949652 |
| *A. cirratum* | 14 | **Mangataipa, Northland** | -35.2482, 173.5351 | WELT SP103514 | S | KT949749 | KT949513 | KT949868 | KT949630 |
| *A. cirratum* | 14 | **Mangataipa, Northland** | -35.2482, 173.5351 | WELT SP103514 | S | KT949750 | KT949514 | KT949869 | KT949631 |
| *A. cirratum* | 15 | **Waimamaku River, Northland** | -35.5843, 173.4179 | AK 316310 | Q | KT949745 | KT949509 | KT949864 | KT949626 |
| *A. cirratum* | 16 | **Waipoua River, Northland** | -35.6351, 173.5063 | AK 316323 | Q | KT949746 | KT949510 | KT949865 | KT949627 |
| *A. cirratum* | 17 | **Maunganui Bluff, Northland** | -35.7783, 173.5703 | AK 308946 | I | KT949725 | KT949489 | KT949844 | KT949606 |
| *A. cirratum* | 18 | **Maungaraho, Northland** | -36.0205, 173.9727 | AK 309439 | A | KT949662 | KT949426 | KT949781 | KT949543 |
| *A. cirratum* | 19 | **Whangaruru North Scenic Reserve, Northland** | -34.3744, 174.3705 | WELT SP103532 | O | KT949740 | KT949504 | KT949859 | KT949621 |
| *A. cirratum* | 20 | **Matapouri Bay, Northland** | -35.5623, 174.5094 | WELT SP103515 | O | KT949741 | KT949505 | KT949860 | KT949622 |
| *A. cirratum* | 20 | **Matapouri Bay, Northland** | -35.5623, 174.5094 | WELT SP103515 | O | KT949742 | KT949506 | KT949861 | KT949623 |
| *A. cirratum* | 21 | **Bream Head, Northland** | -35.8510, 174.5821 | WELT SP103530 | G | KT949720 | KT949484 | KT949839 | KT949601 |
| *A. cirratum* | 21 | **Bream Head, Northland** | -35.8510, 174.5821 | WELT SP103530 | G | KT949721 | KT949485 | KT949840 | KT949602 |
| *A. cirratum* | 22 | **Needle Rocks, Great Barrier Is.** | -36.2378, 175.4845 | AK 311960 | R | KT949747 | KT949511 | KT949866 | KT949628 |
| *A. cirratum* | 23 | **Memory Is., Great Barrier Is.** | -36.2595, 175.4896 | AK 311965 | R | KT949748 | KT949512 | KT949867 | KT949629 |
| *A. cirratum* | 23 | **Raupuke Pt, Great Barrier Is.** | -36.0012, 175.3588 | AK 311980 | X | KT949758 | KT949522 | KT949877 | KT949639 |
| *A. cirratum* | 24 | **Tryphena point, Great Barrier Is.** | -36.3313, 175.4745 | AK 311952 | N | KT949739 | KT949503 | KT949858 | KT949620 |
| *A. cirratum* | 25 | **Aotea Cliffs, Shelly Bay, Auckland** | -36.5713, 174.373 | AK 308963 | I | KT949724 | KT949488 | KT949843 | KT949605 |
| *A. cirratum* | 26 | **Te Henga, Auckland** | -36.8924, 174.4437 | WELT SP103519 | E | KT949712 | KT949476 | KT949831 | KT949593 |
| *A. cirratum* | 26 | **Te Henga,**  **Auckland** | -36.8924, 174.4437 | WELT SP103521 | E | KT949713 | KT949477 | KT949832 | KT949594 |
| *A. cirratum* | 27 | **Waitakere Ranges, Auckland** | -36.9544, 174.6035 | - | E | KT949715 | KT949479 | KT949834 | KT949596 |
| *A. cirratum* | 28 | **Orpheus Bay, Auckland** | -37.0059, 174.5732 | AK 312232 | U | KT949752 | KT949516 | KT949871 | KT949633 |
| *A. cirratum* | 29 | **Maioro, Waikato** | -37.3366, 174.6848 | - | E | KT949714 | KT949478 | KT949833 | KT949595 |
| *A. cirratum* | 30 | **Limestone Downs, Waikato** | -37.4575, 174.7463 | - | E | KT949716 | KT949480 | KT949835 | KT949597 |
| *A. cirratum* | 30 | **Limestone Downs, Waikato** | -37.4575, 174.7463 | - | E | KT949717 | KT949481 | KT949836 | KT949598 |
| *A. cirratum* | 31 | **Papanui Point, Waikato** | -37.8898, 174.7636 | CHR 473343 | A | KT949664 | KT949428 | KT949783 | KT949545 |
| *A. cirratum* | 31 | **Papanui Point, Waikato** | -37.8898, 174.7636 | CHR 473343 | A | KT949665 | KT949429 | KT949784 | KT949546 |
| *A. cirratum* | 31 | **Papanui Point, Waikato** | -37.8898, 174.7636 | CHR 473343 | A | KT949666 | KT949430 | KT949785 | KT949547 |
| *A. cirratum* | 32 | **Waitemata Harbour, Auckland** | -36.8225, 174.7032 | AK 308834 | T | KT949751 | KT949515 | KT949870 | KT949632 |
| *A. cirratum* | 33 | **Waihoka, Otakawhe Bay, Waiheke Island** | -36.8425, 175.1366 | AK 310003 | C | KT949694 | KT949458 | KT949813 | KT949575 |
| *A. cirratum* | 34 | **South of Sandy Bay, Coromandel** | -36.5281, 175.4624 | WELT SP103526 | F | KT949718 | KT949482 | KT949837 | KT949599 |
| *A. cirratum* | 34 | **South of Sandy Bay, Coromandel** | -36.5281, 175.4624 | WELT SP103526 | F | KT949719 | KT949483 | KT949838 | KT949600 |
| *A. cirratum* | 35 | **Te Huruhuru Pt, Pauanui, Coromandel** | -37.0047, 175.8676 | - | C | KT949701 | KT949465 | KT949820 | KT949582 |
| *A. cirratum* | 36 | **Hauturu Island, Whangamata** | -37.2200, 175.8888 | AK 308785 | K | KT949732 | KT949496 | KT949851 | KT949613 |
| *A. cirratum* | 37 | **Rapatiotio Pt, Waihi Beach** | -37.3896, 175.9368 | AK 310541 | K | KT949733 | KT949497 | KT949852 | KT949614 |
| *A. cirratum* | 38 | **Tauwhare Pa Scenic Reserve, Bay of Plenty** | -37.9830, 177.0691 | WELT SP103522 | B | KT949667 | KT949431 | KT949786 | KT949548 |
| *A. cirratum* | 38 | **Tauwhare Pa Scenic Reserve, Bay of Plenty** | -37.9830, 177.0691 | WELT SP103522 | B | KT949668 | KT949432 | KT949787 | KT949549 |
| *A. cirratum* | 38 | **Tauwhare Pa Scenic Reserve, Bay of Plenty** | -37.9830, 177.0691 | WELT SP103522 | B | KT949669 | KT949433 | KT949788 | KT949550 |
| *A. cirratum* | 39 | **Motu, East Cape** | -37.8679, 177.6085 | AK 311292 | B | KT949670 | KT949434 | KT949789 | KT949551 |
| *A. cirratum* | 39 | **Motu, East Cape** | -37.8679, 177.6085 | AK 311292 | B | KT949680 | KT949444 | KT949799 | KT949561 |
| *A. cirratum* | 39 | **Motu, East Cape** | -37.8679, 177.6085 | AK 311292 | B | KT949681 | KT949445 | KT949800 | KT949562 |
| *A. cirratum* | 39 | **Motu, East Cape** | -37.8679, 177.6085 | AK 311292 | B | KT949682 | KT949446 | KT949801 | KT949563 |
| *A. cirratum* | 40 | **Haparapara River, East Cape** | -37.7929, 177.6679 | AK 311376 | J | KT949730 | KT949494 | KT949849 | KT949611 |
| *A. cirratum* | 40 | **Haparapara River, East Cape** | -37.7929, 177.6679 | AK 311376 | J | KT949731 | KT949495 | KT949850 | KT949612 |
| *A. cirratum* | 40 | **Haparapara River, East Cape** | -37.7929, 177.6679 | AK 311376 | J | KT949726 | KT949490 | KT949845 | KT949607 |
| *A. cirratum* | 41 | **Waikawa Stream, East Cape** | -37.6783, 177.7483 | WELT SP104032 | AB | KT949761 | KT949525 | KT949880 | KT949642 |
| *A. cirratum* | 41 | **Waikawa Stream, East Cape** | -37 6783, 177.7483 | WELT SP104032 | AB | KT949762 | KT949526 | KT949881 | KT949643 |
| *A. cirratum* | 41 | **Waikawa Stream, East Cape** | -37 6783, 177.7483 | WELT SP104032 | AB | KT949763 | KT949527 | KT949882 | KT949644 |
| *A. cirratum* | 42 | **Whanarua Bay, East Cape** | -37.6762, 177.7867 | AK 311399 | AC | KT949768 | KT949532 | KT949887 | KT949649 |
| *A. cirratum* | 42 | **Whanarua, East Cape** | -37.6762, 177.7867 | AK 311399 | AB | KT949764 | KT949528 | KT949883 | KT949645 |
| *A. cirratum* | 42 | **Whanarua, East Cape** | -37.6762, 177.7867 | AK 311399 | AB | KT949765 | KT949529 | KT949884 | KT949646 |
| *A. cirratum* | 42 | **Whanarua, East Cape** | -37.6762, 177.7867 | AK 311399 | AB | KT949766 | KT949530 | KT949885 | KT949647 |
| *A. cirratum* | 42 | **Whanarua, East Cape** | -37.6762, 177.7867 | AK 311399 | AB | KT949767 | KT949531 | KT949886 | KT949648 |
| *A. cirratum* | 43 | **Tohora Pirau, East Cape** | -37.5515, 178.1589 | AK 311396 | AC | KT949769 | KT949533 | KT949888 | KT949650 |
| *A. cirratum* | 44 | **Otiki Hill, East Cape** | -37.6870, 178.5423 | AK 311425 | C | KT949696 | KT949460 | KT949815 | KT949577 |
| *A. cirratum* | 44 | **Hicks Bay, East Cape** | -37.5683, 178.2866 | AK 311414 | C | KT949697 | KT949461 | KT949816 | KT949578 |
| *A. cirratum* | 45 | **Onepoto, Hick's Bay, East Cape** | -37.5922, 178.2931 | AK 311424 | C | KT949695 | KT949459 | KT949814 | KT949576 |
| *A. cirratum* | 46 | **Lake Rotoehu, Bay of Plenty** | -38.0323, 176.5156 | WELT SP103528 | M | KT949736 | KT949500 | KT949855 | KT949617 |
| *A. cirratum* | 46 | **Lake Rotoehu, Bay of Plenty** | -38.0323, 176.5156 | WELT SP103528 | M | KT949737 | KT949501 | KT949856 | KT949618 |
| *A. cirratum* | 46 | **Lake Rotoehu, Bay of Plenty** | -38.0323, 176.5156 | WELT SP103528 | M | KT949738 | KT949502 | KT949857 | KT949619 |
| *A. cirratum* | 47 | **Lake Okataina, Bay of Plenty** | -38.1385, 176.4019 | WELT SP103539 | J | KT949727 | KT949491 | KT949846 | KT949608 |
| *A. cirratum* | 47 | **Lake Okataina, Bay of Plenty** | -38.1385, 176.4019 | WELT SP103539 | J | KT949728 | KT949492 | KT949847 | KT949609 |
| *A. cirratum* | 47 | **Lake Okataina, Bay of Plenty** | -38.1385, 176.4019 | WELT SP103539 | J | KT949729 | KT949493 | KT949848 | KT949610 |
| *A. cirratum* | 48 | Kairakau, Hawke’s Bay | -39.9333, 176.9333 | WELT SP103540 | B | KT949671 | KT949435 | KT949790 | KT949552 |
| *A. cirratum* | 48 | Kairakau, Hawke’s Bay | -39.9333, 176.9333 | WELT SP103540 | B | KT949672 | KT949436 | KT949791 | KT949553 |
| *A. cirratum* | 48 | Kairakau, Hawke’s Bay | -39.9333, 176.9333 | WELT SP103540 | B | KT949673 | KT949437 | KT949792 | KT949554 |
| *A. cirratum* | 49 | Tora, Wairarapa | -41.5088, 175.5060 | WELT SP103372 | B | KT949676 | KT949440 | KT949795 | KT949557 |
| *A. cirratum* | 49 | Tora, Wairarapa | -41.5088, 175.5060 | WELT SP103371 | B | KT949677 | KT949441 | KT949796 | KT949558 |
| *A. cirratum* | 49 | Tora, Wairarapa | -41.5088, 175.5060 | WELT SP103371 | B | KT949678 | KT949442 | KT949797 | KT949559 |
| *A. cirratum* | 49 | Tora, Wairarapa | -41.5088, 175.5060 | WELT SP103371 | B | KT949679 | KT949443 | KT949798 | KT949560 |
| *A. cirratum* | 50 | Wainuiomata River Mouth, Wellington | -41.4075, 174.8883 | AK 313242 | B | KT949674 | KT949438 | KT949793 | KT949555 |
| *A. cirratum* | 50 | Wainuiomata River Mouth, Wellington | -41.4075, 174.8883 | AK 313242 | B | KT949675 | KT949439 | KT949794 | KT949556 |
| *A. cirratum* | 51 | Miramar Peninsula, Wellington | -41.2968, 174.8258 | WELT SP103535 | C | KT949692 | KT949456 | KT949811 | KT949573 |
| *A. cirratum* | 51 | Miramar Peninsula,  Wellington | -41.2968, 174.8258 | WELT SP103535 | C | KT949693 | KT949457 | KT949812 | KT949574 |
| *A. cirratum* | 52 | Paekakariki, Wellington | -40.9900, 174.952 | WELT SP103369 | C | KT949702 | KT949466 | KT949821 | KT949583 |
| *A. cirratum* | 52 | Paekakariki, Wellington | -40.9900, 174.952 | WELT SP103369 | C | KT949703 | KT949467 | KT949822 | KT949584 |
| *A. cirratum* | 53 | Puangiangi, Marlborough Sounds | -40.7663,  173.9812 | WELT SP104043 | C | KT949708 | KT949472 | KT949827 | KT949589 |
| *A. cirratum* | 54 | Titirangi Bay, Marlborough Sounds | -41.0194, 174.1338 | WELT SP103708 | C | KT949704 | KT949468 | KT949823 | KT949585 |
| *A. cirratum* | 54 | Titirangi Bay, Marlborough Sounds | -41.0194, 174.1338 | WELT SP103708 | C | KT949705 | KT949469 | KT949824 | KT949586 |
| *A. cirratum* | 54 | Titirangi Bay, Marlborough Sounds | -41.0194, 174.1338 | WELT SP103708 | C | KT949706 | KT949470 | KT949825 | KT949587 |
| *A. cirratum* | 54 | Titirangi Bay, Marlborough Sounds | -41.0194, 174.1338 | WELT SP103708 | C | KT949707 | KT949471 | KT949826 | KT949588 |
| *A. cirratum* | 55 | The Snout, Picton, Marlborough Sounds | -41.2684, 174.0265 | WELT SP103536 | C | KT949698 | KT949462 | KT949817 | KT949579 |
| *A. cirratum* | 55 | The Snout, Picton, Marlborough Sounds | -41.2684, 174.0265 | WELT SP103537 | C | KT949699 | KT949463 | KT949818 | KT949580 |
| *A. cirratum* | 55 | The Snout, Picton, Marlborough Sounds | -41.2684, 174.0265 | WELT SP103537 | C | KT949700 | KT949464 | KT949819 | KT949581 |
| *A. cirratum* | 56 | Ocean Bay, Marlborough Sounds | -41.3319, 174.1011 | WELT SP103442 | C | KT949683 | KT949447 | KT949802 | KT949564 |
| *A. cirratum* | 56 | Ocean Bay, Marlborough Sounds | -41.3319, 174.1011 | WELT SP103442 | C | KT949684 | KT949448 | KT949803 | KT949565 |
| *A. cirratum* | 56 | Ocean Bay, Marlborough Sounds | -41.3319, 174.1011 | WELT SP103442 | C | KT949685 | KT949449 | KT949804 | KT949566 |
| *A. cirratum* | 57 | Abel Tasman Track, Wainui Bay | -40.8057, 172.9532 | WELT SP103524 | C | KT949686 | KT949450 | KT949805 | KT949567 |
| *A. cirratum* | 57 | Abel Tasman Track, Wainui Bay | -40.8057, 172.9532 | WELT SP103524 | C | KT949687 | KT949451 | KT949806 | KT949568 |
| *A. cirratum* | 58 | Hanson Winter Scenic Reserve, Golden Bay | -40.8334, 172.8888 | WELT SP103523 | C | KT949688 | KT949452 | KT949807 | KT949569 |
| *A. cirratum* | 58 | Hanson Winter Scenic Reserve, Golden Bay | -40.8334, 172.8888 | WELT SP103523 | C | KT949689 | KT949453 | KT949808 | KT949570 |
| *A. cirratum* | 58 | Hanson Winter Scenic Reserve, Golden Bay | -40.8334, 172.8888 | WELT SP103531 | C | KT949690 | KT949454 | KT949809 | KT949571 |
| *A. cirratum* | 58 | Hanson Winter Scenic Reserve, Golden Bay | -40.8334, 172.8888 | WELT SP103531 | C | KT949691 | KT949455 | KT949810 | KT949572 |
| *A. candidum* |  | Paynes Ford, Golden Bay | -40.8873, 172.8122 | WELT SP103527 | - | KT949772 | KT949417 | KT949891 | KT949653 |
| *A. candidum* |  | Lake Wakatipu | -40.0424,  168.63704 | - | - | KT949773 | KT949418 | KT949892 | KT949654 |
